# Natural products for Gut-X axis: pharmacology, toxicology and microbiology in mycotoxin-caused diseases

**DOI:** 10.3389/fphar.2024.1419844

**Published:** 2024-06-19

**Authors:** Kaiqi Li, Shiqi Wang, Wuyi Qu, Abdelkareem A. Ahmed, Wael Enneb, Mohammad Diya’ Obeidat, Hao-Yu Liu, Tadelle Dessie, In Ho Kim, Saber Y. Adam, Demin Cai

**Affiliations:** ^1^ College of Animal Science and Technology, Yangzhou University, Yangzhou, China; ^2^ Department of Veterinary Biomedical Sciences, Botswana University of Agriculture and Agriculture and Natural Resources, Gaborone, Botswana; ^3^ Department of Animal Production, Jordan University of Science and Technology, Irbid, Jordan; ^4^ Jiangsu Key Laboratory of Animal Genetic Breeding and Molecular Design, College of Animal Science and Technology, Yangzhou University, Yangzhou, China; ^5^ International Livestock Research Institute, Addis Ababa, Ethiopia; ^6^ Department of Animal Resource and Science, Dankook University, Cheonan, Republic of Korea

**Keywords:** mycotoxin, mycotoxin-induced diseases, natural products, gut-x axis, gut microbial modulation

## Abstract

**Introduction:** The gastrointestinal tract is integral to defending against external contaminants, featuring a complex array of immunological, physical, chemical, and microbial barriers. Mycotoxins, which are toxic metabolites from fungi, are pervasive in both animal feed and human food, presenting substantial health risks.

**Methods:** This review examines the pharmacological, toxicological, and microbiological impacts of natural products on mycotoxicosis, with a particular focus on the gut-x axis. The analysis synthesizes current understanding and explores the role of natural products rich in polysaccharides, polyphenols, flavonoids, and saponins.

**Results:** The review highlights that mycotoxins can disrupt intestinal integrity, alter inflammatory responses, damage the mucus layer, and disturb the bacterial balance. The toxins' effects are extensive, potentially harming the immune system, liver, kidneys, and skin, and are associated with serious conditions such as cancer, hormonal changes, genetic mutations, bleeding, birth defects, and neurological issues. Natural products have shown potential anticancer, anti-tumor, antioxidant, immunomodulatory, and antitoxic properties.

**Discussion:** The review underscores the emerging therapeutic strategy of targeting gut microbial modulation. It identifies knowledge gaps and suggests future research directions to deepen our understanding of natural products' role in gut-x axis health and to mitigate the global health impact of mycotoxin-induced diseases.

## 1 Introduction

Mycotoxins, which are low molecular weight (MW around 700 Da) secondary metabolites primarily produced by Aspergillus, Penicillium, and Fusarium, are highly toxic to humans and animals (Liew and Mohd-Redzwan, 2018). Their impact on health is influenced by the type of mycotoxin, exposure levels and duration, physiological and nutritional status, and potential interactions with other substances (Gajęcka et al., 2013). Genetic variability means some individuals are more susceptible to mycotoxicosis due to gene mutations in the cytochrome P450 (CYP450) gene, affecting mycotoxin metabolism (Sun et al., 2016). The FAO estimates that mycotoxins contaminate about 25% of global agricultural production, leading to an annual loss of 50 million tons of food (Iheshiulor et al., 2011). However, the actual prevalence may be as high as 60%–80% (Eskola et al., 2020). Mycotoxins can cause a range of severe health effects, including immunotoxicity, organ damage, cancer promotion, and neurological harm (Romina and Romina Alina, 2022).

Mycotoxins can cause a range of severe health effects, including immunotoxicity, organ damage, cancer promotion, and neurological harm (Rajakovich and Balskus, 2019). Disruptions in gut microbiota, or dysbiosis, have been linked to various diseases (Fung et al., 2017). Many human and animal studies have shown that dysbiosis of gut microbiota is linked with diseases as asthma, autism, colon cancer, Crohn’s disease, cardiovascular disease, diabetes, food allergies, eczema, irritable bowel syndrome, hepatic encephalopathy, obesity, and mental disorders (Korem et al., 2015). External factors like toxins, diet, and medication can alter gut microbiota composition (Izco et al., 2021). Some bacteria can metabolize or bind to mycotoxins, protecting against their harmful effects, but mycotoxins can also have adverse effects on the gut microbiota, such as affecting the composition at various levels of the community, threatening the colonization of beneficial bacteria, and promoting the growth of harmful bacteria (Izco et al., 2021). However, not much research has been conducted on how mycotoxins affect the gut microbiome (Robert et al., 2017; Liew and Mohd-Redzwan, 2018). The gut microbiota axis (GMA) is a vital communication network between the gut and other organs (Sampson and Mazmanian Sarkis, 2015). Studies on the microbiome have shed light on the interactions between the gut and other systems like the liver, brain, and lungs (Shi et al., 2023).

Chinese herbal medicine (CHM), known for its antioxidant, immunomodulatory, anti-tumor, and antifungal properties, is widely used to prevent and treat metabolic diseases (Ding et al., 2023). Common natural antioxidants mostly contain flavonoids, polyphenols, polysaccharides, and saponins as their active metabolites, which work as antioxidants by inhibiting the synthesis of lipid reactive oxygen species (ROS) and simultaneously turning H_2_O_2_ into H_2_O (Huang et al., 2017; Wang et al., 2022). These extracts contain natural antioxidants that are safer and more environmentally friendly than synthetic ones (Ates and Ortatatli, 2021). Antioxidant botanical drugs such as curcumin, lycopene, fucoidan, and artemisinin have been used widely in recent years to prevent mycotoxin exposure and disorders related to ferroptosis (Li et al., 2021; Wu et al., 2022). Some CHM preparations are widely used to treat cancer due to their properties of invigorating vital energy and anti-cancer characteristics, primarily by resisting inflammatory responses and enhancing immune function (Wang et al., 2020). The aim of this study was to discuss the pharmacological, toxicological and microbiological effects of natural products on gut-x axis in mycotoxin-caused diseases to provide novel insight into future therapies.

## 2 Gut health, microbiota and mycotoxins

In general, the GI tract’s intestinal barrier serves as a filter for eliminating harmful mycotoxins. Nevertheless, it has been found that a number of mycotoxins cause harm to the gastrointestinal tract. For example, mycotoxins may cause disruption with the normal functioning of the intestinal barrier and absorption of nutrients. Certain mycotoxins also affect the histomorphology of intestine (Liew and Mohd-Redzwan, 2018). However, the liver and the digestive tract are both sites of mycotoxin metabolism. Liver is responsible for detoxification that following mycotoxin exposure, as the central metabolic site (Zong et al., 2022). The adverse effects of mycotoxins within the GIT may be restricted by intestinal metabolism, which can take place in the gut epithelium or by gut microbes. This is particularly true for ruminants, who may convert a variety of mycotoxins into metabolites that are non-toxic. It has been suggested that the microbial population in the rumen has a detoxifying role in ruminant resistance to some mycotoxins. Many compounds, like ZEA, DON, or OTA, are efficiently made non-toxic by the microorganisms of the rumen prior to absorption (Cavret and Lecoeur, 2006).

### 2.1 Aflatoxin (AFB)

The animal mostly gets into contact with AFB through ingestion, and the gastrointestinal tract’s microbiota is first exposed (Huang et al., 2023). The small intestine is the main site of AFB absorption, with the duodenum being the intestinal location with the highest absorption efficiency (Sayin et al., 2013). AFB1 exposure resulted in intestinal diseases such as villi degeneration and the formation of sub-epithelial space in the duodenum and ileum. Exposure to AFB1 can have negative effects on the gut, such as immune system problems, intestinal barrier degradation, cell proliferation, and apoptosis. Compared to the other mycotoxins, AFB1 is the most life-threatening mycotoxin affected the gut (Liew and Mohd-Redzwan, 2018). The impact of AFB1 on the gut microbiota was evaluated by extracting the contents of the broiler chickens’ duodenum, following AFB1 exposure and using 16S rRNA sequencing to analyze the changes in abundance and diversity of the gut microbiota (Huang et al., 2023). AFB1 at a dosage level of 1 ppm significantly (*p* < 0.05) decreased total LAB in the broiler, according to a recent study. On the other hand, the broiler group offered 1.5 and 2 ppm of AFB1 showed a significant (*p* < 0.05) increase in the total number of Gram-negative bacteria and LAB. Additionally, it has been observed that broilers exposed to 2.5 ppm of AFB1 produced more total volatile fatty acids, indicating an association between higher AFB1 dose and a higher incidence of LAB in the gut (Galarza-Seeber et al., 2016).

### 2.2 Fusarium graminearum (T-2, DON)

Fusarium graminearum is the main fungi species that produces tricothecenes and Deoxynivalenol (DON). DON’s effect is often dependent on dose- and/or exposure time, and can cause severe damage to the gastrointestinal tract, liver, and other metabolic and immunity-related organs (Pestka, 2010). Furthermore, the histological analysis provided features such as nuclear vacuolation, neutrophil infiltration, and dilated and congested blood vessels, confirming liver damage (Wang et al., 2023). Oxidative stress has been a serious issue involved in DON-induced hepatotoxicity that leads to overproduce of ROS, and extreme ROS could trigger lipid peroxidation (LPO) and damage cellular macromolecule function (Wang et al., 2023). Using porcine epithelial cell, trichothecenes improved intestinal permeability by the downregulation of tight junction protein expression (Osselaere et al., 2013). Furthermore, in animal treated with trichothecenes, previous studies showed a significant (*p* < 0.05) decrease in the number of goblet cells secreting mucin. Mucin is mainly involved in the gut barrier function. Furthermore, trichothecenes have been linked to a reduced intestinal level of IL-8, which is in charge of eliminating pathogens (Kadota et al., 2013). Generally, trichothecenes exert negative impacts on GI tracts specifically on the gut absorption, immunity, and integrity (Liew and Mohd-Redzwan, 2018). It was demonstrated that giving T-2 toxin to pigs for 1 week was enough to substantially increase the number of aerobic bacteria in their intestines (Verbrugghe et al., 2012). Trichothecene has been demonstrated to have an important effect on bacterial populations; however, the exact mechanism causing this alteration is still not completely understood.

### 2.3 Zearalenone (ZEA)

Zearalenone (ZEA) was shown to accelerate cell migration, increase colony formation, and enhance cell proliferation in a colon cancer cell line HC-116 (Abassi et al., 2016). Additionally, ZEA has been demonstrated to downregulate the expression of tumor-suppressor genes particularly (PCDH11X, DKK1, and TC5313860) in intestinal cells (Taranu et al., 2015). In fact, the carcinogenic effects of ZEA were caused by the modification of gene expression. An *in vitro* study revealed that the human gut bacteria transformed zearalenone molecules into unknown metabolites (Gratz et al., 2017). The alterations in the gut microbiota were assessed using the Biolog-Eco Plate method, which allows only the quantification of culturable bacteria. After 6 weeks of ZEA application, the findings demonstrated a significant (*p* < 0.05) decrease in the concentrations of *E. coli*, Enterobacteriaceae, and *Clostridium perfringens*. (Piotrowska et al., 2014).

### 2.4 Fumonisins (FB)

By using intestinal cell lines (IPEC-1, Caco-2, and HT29), it was discovered that FB1 reduced cell viability and proliferation in a concentration-dependent manner (Minervini et al., 2014). A potential mechanism has been proposed through the accumulation of sphinganine by FB1. Sphinganine accumulation in intestinal epithelial cells inhibited the G0/G1 phase of the cell, resulting in to growth inhibition and apoptosis (Angius et al., 2015). Furthermore, FB1 altered the integrity of the intestinal barrier by decreasing the expression of the tight junction (TJ) protein (Romero et al., 2016). Besides, elevated FB1 levels also caused growth in the intestinal goblet cells of pigs and broilers. Mucin secretion is known to be elevated in goblet-cell hyperplasia. On the other hand, continuous mucin hypersecretion may reduce the quantity of goblet cells, which would destroy the mucus barrier (Johansson and Hansson, 2016). Upon LPS exposure to the FB1-treated cell line, a decrease in IL-8 synthesis has been detected (Brazil et al., 2013). FB1 often resulted in immune dysfunction, reduced intestinal barrier function, and increased intestinal cell death in the gut. By using capillary electrophoresis single-stranded conformation polymorphism (CE-SSCP), it is demonstrated that fumonisins reduced the fecal microbiota SSCP-profiles similarity of the fumonisins treated swine, in comparison to the untreated control group. The findings indicated that the variety of the microbiota is increasing (Burel et al., 2013).

### 2.5 Ochratoxin (OTA)

Studies conducted *in vitro* demonstrated that OTA reduced the SGLT1 transporter’s ability to absorb glucose (Peraica et al., 2011). In addition, compared to control animals, OTA-treated animals developed more quickly and harmful parasite infections in chicks and turkeys (which is caused by Eimeria acervulina and E. adenoeides). The finding revealed that animals fed with OTA had developed lesion and oocyst indexes in the intestine and more damage at mucosa (Manafi et al., 2011). It has been found that the oxidative stress caused by OTA is related to the intestine IPEC-J2 cells’ apoptosis (Wang H. et al., 2017). Inflammation pathway in the intestine was also affected by OTA. Piglets exposed to the toxin revealed a significant (*p* < 0.05) decrease in the expression of inflammation-related cytokines, including IL-8, IL-6, IL-17A, IL-12, and IL-18, in their intestines (Marin et al., 2017). OTA affected the gut through decreasing the absorption of nutrients, disrupting the permeability of the intestinal lining, causing cell apoptosis, and modifying the immune system (Liew and Mohd-Redzwan, 2018). An *in vivo* study on rats showed that the gut microbiota’s diversity was reduced by OTA treatment. Moreover, at the genus level the OTA increases the quantity of *Lactobacillus* and decreased the populations of *Bacteroides*, Dorea, *Escherichia*, Oribacterium, Ruminococcus, and Syntrophococcus. The findings showed that *lactobacillus* was more resistant to OTA and that it might be involved in the process of OTA detoxification. Additionally, it was reported that the OTA treatment has positive effects on facultative anaerobes (Guo et al., 2014). This may indicate that the OTA could alter the gut microbiota in a way that is harmful to the host’s health.

## 3 Gut-liver axis and mycotoxin

**FIGURE 1:**
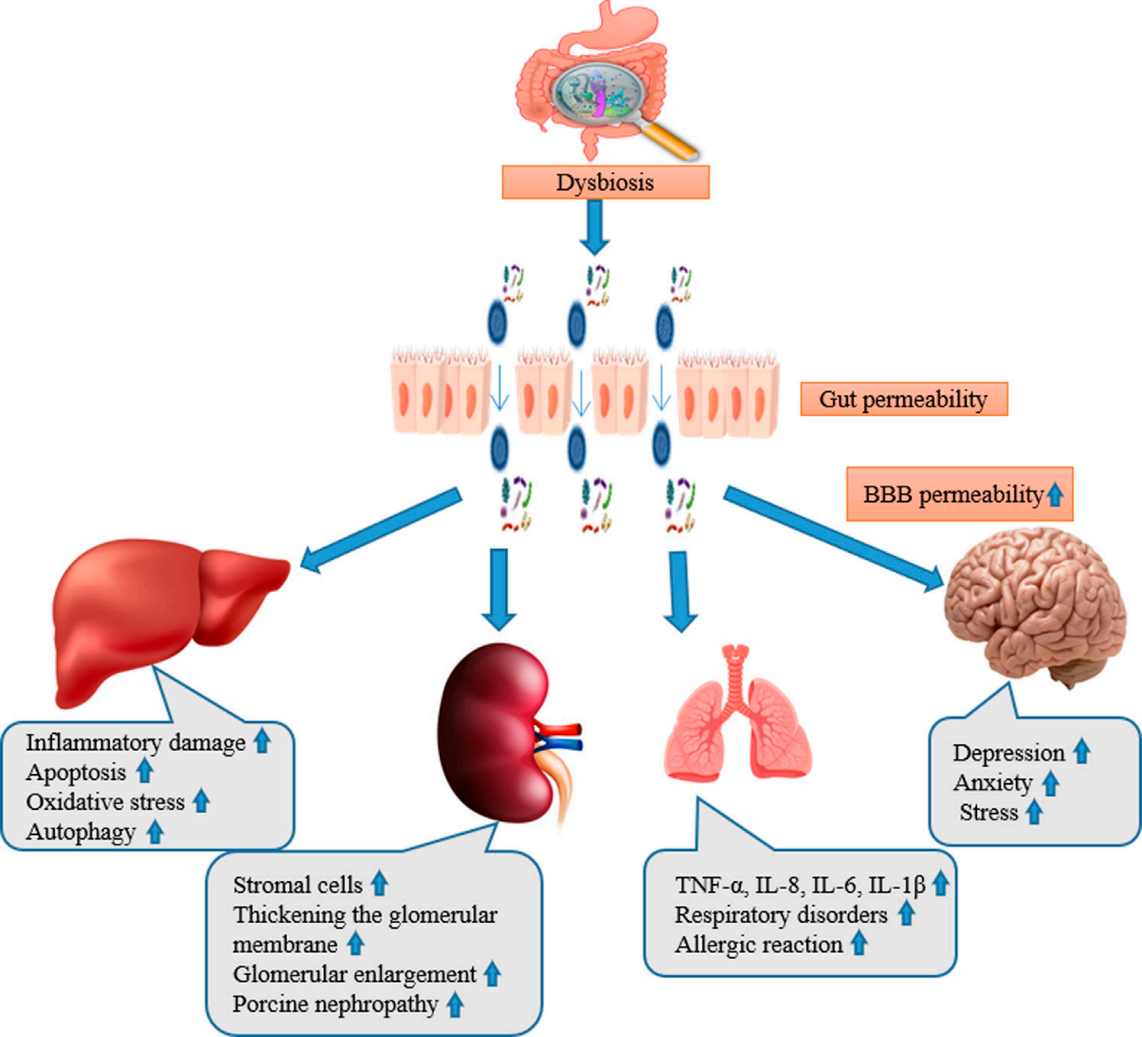
The abstract of gut liver, kidney, lung, and brain axis and their main pathological alteration during mycotoxicosis.

There exists a delicate connection between gut and liver for their functional and anatomical familiarity (Luo and Zhou, 2021). Gut dysbiosis has been related with the pathogenesis of a wide spectrum of liver diseases including autoimmune hepatitis, autoimmune cholangiopathies, alcoholic liver disease, non-alcoholic liver disease (NAFLD), hepatocellular carcinoma, acute liver injury, and liver fibrosis/cirrhosis (Hartmann et al., 2019). Aflatoxin B1, DON, fumonisin B1, ochratoxin A, and T2 are among the mycotoxins that have been shown in multiple studies to increase the permeability of the intestinal epithelium in many animals (Alassane-Kpembi et al., 2019). This is mainly the consequence of protein synthesis being inhibited. The passage of antigens into the bloodstream increases as a result. This makes the animal more susceptible to infectious intestinal diseases. Moreover, mycotoxins are absorbed more quickly due to the damage they cause to the intestinal barrier. Aflatoxin toxicity is mostly aimed towards the liver through entero-hepatic circulation after absorption. For example, AFB1 can induce inflammatory damage, apoptosis, oxidative stress, and autophagy to cause liver function damage (Zhang et al., 2021; Lin et al., 2022) (Figure 1).

## 4 Gut-lung axis and mycotoxin

Both the gastrointestinal and respiratory systems are physically close to one another, and they also have similar embryonic origins and structures, which raises the possibility that their interactions are multifaceted. (Haldar et al., 2023). Aspiration may allow the microbiota from the gut to enter the lungs, exposing the respiratory tract’s epithelial surface to a variety of microorganisms (Hartman et al., 2010). The lung is a functional organ for gas exchange, the interaction between the gut microbiota and nutrition is associated with respiratory disorders, such as asthma, chronic obstructive pulmonary disease (COPD), and allergies (Segal et al., 2016). The lungs’ inflammatory response is impeded by short chain fatty acids generated from gut bacteria (Barcik et al., 2020). In addition, gut bacteria have the ability to produce anti-inflammatory active metabolites such biogenic amines, that are represented by histamine (Pugin et al., 2017) and oxylipins (Levan et al., 2019). Research indicates that various microbes, viruses, changes in the microbiome, stress, exposure to the environment, and dietary modifications can all have an effect on the lungs (Mims, 2015), and they are risk factors for such diseases. The majority of experimental studies on lung disorders has only discussed how the gut microbiota contributes to the development of lung disorders. Longitudinal studies are required to correlate the severity of chronic lung diseases with changes in the gut microbiome (Shi et al., 2023). DON has been demonstrated to have a distinct cytotoxic effect on human primary cells. Collagens I, III, and IV, fibronectin secretion, apoptotic and necrotic cell death, and cell viability were all evaluated. A decrease in viability can be observed in 2 cell types, with fibroblasts reacting more sensitive than epithelial cells. Additionally, fibroblasts treated with DON mostly experienced apoptotic cell death (Königs et al., 2007) (Figure 1).

## 5 Gut-brain axis and mycotoxin

Maintaining the host’s healthy mental state, particularly brain function, depends on the mutualistic relationship that exists between the gut microbiota and the brain (Mohajeri et al., 2018; Ganci et al., 2019). In fact, the microbiota-gut-brain axis permits bilateral interaction between the gut microbiota and the central nervous system (CNS) (Cryan et al., 2019). This communication is based on multiple complex pathways, most of which transfer sensory information from the gastrointestinal (GI) tract and then convert it into signals related to hormones, brain activity, and immunity (Pferschy-Wenzig et al., 2022). These signals supplementary transfer information to the central nervous system (CNS) whichever individually or cooperatively (Lach et al., 2018). Three main pathways are thought to be involved in the interactions between the gut microbiota and the brain: 1) direct and indirect signaling through chemical transmitters, such as hormones, neurotransmitters, or microbial metabolites (e.g., short-chain fatty acids, or SCFAs), that can be directly synthesized by the gut microbiota or have their levels modulated by gut microbiota; 2) neural pathways, such as modulation of vagus nerve activity; and 3) immune system signaling, such as microglia-mediated effects or effects of circulating cytokines that can modify the activity of the hypothalamic–pituitary–adrenal (HPA) axis (Cenit et al., 2017; Farzi et al., 2018; Cryan et al., 2019).

Once the mycotoxin passes through the intestinal wall and reaches the plasma, some of them (FUM, DON) may alter the brain functions. Data demonstrates that the neurologic effects of DON are partially dependent on DON’s direct action on brain cells, even though some of these modifications may be related to peripheral effects (Maresca, 2013). This needs the passage of the blood-brain barrier (BBB) by the toxin. Endothelial and glial cells adhere closely to create the blood-brain barrier (BBB) that acts as a selective barrier to restrict the passage of chemicals from the plasma into the cerebrospinal fluid (CSF) (Nico and Ribatti, 2012). The capability of DON to cross the BBB depends on the animal species. In pigs, 25%–30% of the plasmatic DON is found in the CSF, the toxin remaining detectable in the fluid 20 h after ingestion and having a half-life in the CSF similar to its plasmatic one (Maresca, 2013). Nothing is known regarding the mechanism(s) permitting DON to be transported (absorbed and excreted) across the BBB. In conclusion, mycotoxins can cause changes in the gut microbiota, intestinal permeability, neuroinflammation, oxidative stress, and consequent neurological and behavioral symptoms. All of these may disrupt the delicate balance of the gut-brain axis. To improve animals’ general health and wellbeing,such as improving animal immunity to viruses and developing ways to dispose of toxin residues from within. It is still necessary to investigate how these toxins impact the gut-brain axis and how to reduce their effects (Figure 1).

## 6 Gut-kidney axis and mycotoxin

The kidney is considered the metabolic organ of the body, with its main function of removing metabolic waste products and maintaining the equilibrium of the body (Sutherland, 2020). The gut-kidney axis (GKA) is linked with a diversity of kidney diseases, such as kidney stones (Ticinesi et al., 2019), chronic kidney disease (Ramezani et al., 2016), and diabetes (Sircana et al., 2019). The intestinal microflora will change when the alterations in renal function result in excessive ammonia in blood and increased intestinal pH (Shi et al., 2023). These alterations motivate the intestine to reduce mucus manufacture (Vaziri et al., 2012), destructive the intestinal epithelium (Hobby et al., 2019), disturbing the integrity of the intestinal barrier (Plata et al., 2019), changing intestinal permeability (Meijers et al., 2018), and permitting the microflora to be transported to the systemic circulation through the blood to act on the kidneys (Shi et al., 2014). The microbiota in the gut breaks down macromolecules to produce neurotransmitters that have an impact on the kidney (Santisteban et al., 2017). In addition, the gut microbiota can produce neurotransmitters such as acetylcholine, norepinephrine, and gamma-aminobutyric acid (Lyte, 2014), which connect with the metabolically dependent immune pathways through the sympathetic nervous system (SNS) to control the function of the kidney (Al Khodor and Shatat, 2017). The effects of dietary mycotoxin on broiler kidneys include increased stromal cells and thickened of the glomerular basement membrane, glomerular enlargement, cytoplasmic vacuolation of tubular epithelial cells, collapse of the renal glomerulus, and structural damage (Śliżewska et al., 2019; Saleemi et al., 2020), poultry ochratoxicosis, porcine nephropathy, human endemic nephropathies, and urinary tract tumors (Heussner and Bingle, 2015) (Figure 1).

## 7 Effect of natural products on gut-x axis (pharmacology, toxicology and microbiology) during mycotoxicosis

The pharmacological effects of natural metabolites have drawn an enormous amount more attention in recent years (Pan et al., 2019). Clinical data and *in vitro* studies have shown the beneficial effects of natural products on a wide range of diseases (Ennab et al., 2019; Mustafa et al., 2019; Ullah et al., 2020; Ennab et al., 2023). These changes in the gut microbiota caused by natural substances demonstrate that natural products have a preventive effect on the development of inflammation into cancer. Natural products have the ability to control the Firmicutes/Bacteroidetes ratio, and they also increase the number of potentially helpful microorganisms in feces and decrease the relative abundance of potentially pathogenic microbes (Lu et al., 2022). For instance, in HFD-induced obese mice, the ratio of Firmicutes/Bacteroidetes (F/B) reduced to normal levels after receiving a high dose of 300 mg/kg of MDG-1, which was extracted from the root of Ophiopogon japonicas, for a duration of 12 weeks (Shi et al., 2015), declined the number of pathogenic bacteria (*Streptococcus* and *Escherichia coli*) (Wang et al., 2011). Feeding mice 0.5% curcumin for 14 weeks can significantly prevent age-related decreases in alpha diversity, increase bacterial richness, increase the relative abundance of Lactobacillales, and decrease the relative plenty of members of the Coriobacterales order (McFadden et al., 2015), that can utilize strong anti-inflammatory, antioxidative and antiproliferative effects (McFadden et al., 2015). Microbes are vital contributors to the host metabolism, considering that numerous important components, such as vitamin K, indoles, gamma amino butyric acid, folate, and short-chained fatty acids (SCFAs), are produced by microbiota (Brown and Hazen, 2018). These metabolites are generally implicated in many physiological and pathological processes, which may be related to a number of diseases. Natural products may control the microbiome’s metabolism by changing the bacterial structure, which is important in preserving intestinal homeostasis (Ma et al., 2018). Some microbial population (specially the gut bacteria) have a significant detoxification role in the gut-liver cycle during mycotoxin injury (Zhang et al., 2022); therefore, natural products may enhance the capability of the microbial induced mycotoxin resistance.

Mycotoxins can overwhelm the immune system, leading to increased susceptibility to infections. Several natural products have demonstrated immunomodulatory properties, such as echinacea (Echinacea purpurea), ginger (Zingiber officinale), and garlic (Allium sativum), that may increase resistance to mycotoxicosis and enhance the immune response. Natural products modulate the immunological status by modifying the concentration of immune components like IL-22, activating T reg cells, or preventing the development of Th17 cells (Pan et al., 2019). Some natural products can help the body eliminate mycotoxins more effectively since they possess detoxifying properties. For example, it's been discovered that activated charcoal binds to mycotoxins in the gastrointestinal tract, blocking their absorption and promoting their elimination. In addition of the forementioned activities of the natural product, there are many other activities has been demonstrated in previous studies, to reduce mycotoxin toxicity *in vivo* and vitro (Table 1). It is worth noting that, as shown in the table, due to differences in toxin types, species, and methods of administration, there may be significant variation in the effective dosage of natural products. Additionally, the manner in which natural products are administered (gavage, oral intake, injection) to achieve the optimal effect must also be taken into consideration. A considerable amount of experimentation is still required to determine the appropriate intake methods and dosages for practical application in production.

**TABLE 1:** Analysis on the impact of plant extracts and their metabolites on the toxicity caused by mycotoxin.

Plant	Activities agents	Model	Treatment method	Grouping situation	Mode of action
Solanum lycopersicum (tomatoes)	Rutin	Male albino rats((180–200 g, 12 weeks of age)	Incorporated into the feed, 28 days	Control	Reversed T-2 toxin-induced lipid peroxidation in liver homogenate Wu et al. (2017)
				0.1 mg/kg bw T-2 toxin/d	
				0.1 mg/kg bw T-2 toxin +50 mg/kg rutin/d	
	2013Lycopene	Male broiler chicks (7–28 days of age)	Incorporated into the feed, 7 days	Control	Protected against reproductive, inflammatory, hormonal, and oxidative damage induced by OTA and AFB1 in mice, also inhibited T-2 toxin induced oxidative stress Palabiyik et al. (2013); Wu et al. (2017), Hedayati et al. (2019)
				1.5 mg/kg bw T-2 toxin/d	
				25 mg/kg bw lycopene/d	
				1.5 mg/kg bw T-2 toxin +25 mg/kg bw lycopene/d	
		Male Sprague-Dawley rats (<200 g)	Gavaged, 14 days	Control	
				0.5 mg/kg bw OTA toxin/d	
				5 mg/kg bw lycopene/d	
				0.5 mg/kg bw OTA toxin +5 mg/kg bw lycopene/d	
		Adult male Wistar-Albino rats (180–220 g, 12 weeks of age)	Lycopene gavaged, AFB1 toxin intraperitoneally injected, 15 days	Control	
				2.5 mg/kg bw AFB1 toxin/d	
				10 mg/kg bw lycopene/d	
				2.5 mg/kg bw AFB1 toxin +10 mg/kg bw lycopene/d	
	Cyanidin 3-O-beta-D-glucoside(C3G)	In vitro fibroblasts	48 h, 72 h	OTA at 25 and 50 μM	Protected against OTA-induced oxidative stress Do et al. (2015)
				OTA at 25 and 50 μM + C3G at 0.125 and 0.250 mM	
Thymus vulgaris (Thyme)	Thyme oil	Egyptian male sheep	Incorporated into the feed, 2 weeks normal treatment +4 weeks differentiated treatment	Control	Mitigated the liver toxicity caused by AFB and restore the overall performance of the sheep. Abdel-Fattah et al. (2015)
				10 mg/kg AFB toxin/d	
				250/500 mg thyme oil/d	
				10 mg/kg AFB +250/500 mg thyme oil/d	
Curcuma amada (Ginger)	Ginger	Male Wistar rats	Orally treated with GE daily, with the administration of AFB1 alternative day for 28 days	100 mg/kg GE/d + 200 μg/kg AFB1/2d	Inhibited AFB1, STE and PAT toxicity Vipin et al. (2017)
				250 mg/kg GE/d + 200 μg/kg AFB1/2d	
	6-Gingerol	HepG2 cells In vitro	Treated with 6-gingerol (10 μM) for 1 h then exposed to different concentrations of PAT	6-gingerol at 10 μM	Reduced PAT induced DNA damage in HepG2 Yang et al. (2011)
				6-gingerol at 10 μM + PAT at 15 μM	
				6-gingerol at 10 μM + PAT at 30 μM	
				6-gingerol at 10 μM + PAT at 60 μM	
Syzygium aromaticum (Clove)	Clove	In vitro	Added to the growth media of Aspergillus flavus and Aspergillus parasiticus separately	concentration at 0.1%, 0.2% and 0.5%	Inhibit the growth of toxin-producing Aspergillus flavus and Aspergillus parasiticus Hussain (2012)
	Quercetin	In vitro	Added to a reaction system containing 0.1 M Tris-HCl, 43 μM AFB1, 5 mM Mg2+, 0.65 mM NADPH, then add 1 mg of microsomal protein	DMSO control	Prevented AFB1 carcinogenesis Bhattacharya and Firozi (1988)
				1% (v/v) quercetin	
Curcuma longa L. (Turmeric)	Curcumin	Male Fisher-344 rats	Curcumin incorporated into the feed, AFB1 gavaged	Control	Hepatoprotective effects against AFB1 toxicity Nayak and Sashidhar (2010)
				20 µg AFB1/d	
				20 µg AFB1/d + 0.05% (w/w) curcumin/d (for last 3 weeks)	
				0.05% (w/w) curcumin/d	
		Broiler chickens(1–21 days post-hatch)	Incorporated into the feed, 3 weeks	Control	Alleviate oxidative stress caused by AFB1 Nayak and Sashidhar (2010), Limaye et al. (2018)
				444 mg/kg curcumin/d	
				1 mg/kg AFB1/d	
				74 mg/kg curcumin +1 mg/kg AFB1/d	
				222 mg/kg curcumin +1 mg/kg AFB1/d	
				444 mg/kg curcumin +1 mg/kg AFB1/d	
Camellia sinensis L. (green tea) and a variety of plants	Epigallocatechin-3- gallate (EGCG)	HT-29 cells In vitro	EGCG added to cells treated with DON	5–20 μM EGCG +250–1000 ng/mL DON	Inhibited the toxic effects and inflammatory responses induced by DON Kalaiselvi et al. (2013)
Acacia trees	Gum Arabic(GA)	Male Wistar rats	GA dissolved in water, AFB1 dissolved in saline solution, 28 days	Control	Reverse the induced inflammation, oxidative damage, and apoptosis in AF-exposed rats Ahmed et al. (2022)
				7.5 g/kg bw GA/d	
				200 μg/kg bw AFB1/d	
				7.5 g/kg bw GA + 200 μg/kg bw AFB1/d	

The toxicity of medicinal plants and natural products is strictly associated to the presence of bioactive metabolites in the plant substantial and their toxic potential (Woo et al., 2012). The issue becomes considerably more intricate when considering complex, heterogeneous botanical drugs mix which can cause unpredictable effects (Efferth et al., 2012). Many natural products have hepatotoxic, carcinogenic, cytotoxic, genotoxic, phototoxic, possible pulmonary toxicity, embryotoxic, and neurotoxic effects (Wang et al., 2021). Utilizing natural products during mycotoxicosis may have a taxological effect; however, the precise effect will differ based on the natural product used, the dosage, the individual’s susceptibility, and any interactions with other medications or substances.

## 8 Natural products, gut tight junction and regulating of intestinal microbes

Tight junction often serves as a barrier to protect intestinal epithelial cells from bacterial endotoxins (Lu et al., 2022). A high-fat diet (HFD) can increase the gut microbiota-induced production of lipophilic acid synthase (LPS), and this in turn may disrupt the expression of tight junction proteins and ultimately enhance intestinal permeability (Cani et al., 2008). *Flos Lonicera,* is one of the best-known traditional Chinese medicines, has the ability to modify tight junctions at the cellular level by reversing the negative effects of lipopolysaccharides (LPS) and increasing certain microbiota. These actions had a positive impact on preserving the integrity of the intestinal barrier (Wang et al., 2014). Berberine pretreatment has the potential to decrease intestinal permeability and enhance the LPS-induced redistribution of the tight junction-related proteins Claudin-1 and Claudin-4 (Gu et al., 2011). In a previous study, Curcumin has been demonstrated to be able to reduce the disruption of intestinal epithelial barrier functions when its effects were investigated on Caco-2 and HT-29 cells. Curcumin inhibited the release of LPS-secreted IL-1β, stimulated macrophages and IEC, and prevented the disintegration of tight junction proteins, such as ZO-1, Claudin-1, Claudin-7, and actin filaments (Wang J. et al., 2017). All of the aforementioned results demonstrated that tight junction proteins, inflammation, and probiotic abundance can all be positively impacted by natural products on the intestinal barrier (Lu et al., 2022).

## 9 Discussion

Natural products hold significant promise for mitigating mycotoxin-related issues and preserving gut-x axis health. They have exhibited beneficial pharmacological, toxicological, and microbiological properties that can counteract the negative impacts of mycotoxins along the entire gut-x axis. These substances can help balance gut microbiota, regulate gut permeability, boost immunity, alleviate inflammation, and the detoxification of mycotoxins in both animals and humans. Given the complexity of gut-x axis dynamics and host-mycotoxin interactions, in-depth research is essential to establish an effective intervention model including changes in gut microbiota, alterations in metabolites and key targets. This includes exploring cellular processes and molecular mechanisms that enable natural products to enhance gut health and facilitate mycotoxin removal. A thorough toxicological evaluation of these products is necessary to establish their safety profiles, especially regarding potential medication interactions and long-term or high-dose use side effects. Additionally, the economic feasibility of incorporating natural products into the diet, food products, or supplements should also be taken into consideration.

The potential of natural products in treating mycotoxin-induced diseases should be further explored, with a focus on their incorporation into dietary feeds, food products, and supplements. Effective collaboration between researchers and the food industry is crucial for the successful implementation of these natural solutions. By addressing current research gaps and adopting a multidisciplinary approach, our knowledge of how natural products can support gut-x axis health amidst mycotoxin challenges can be significantly enhanced.
